# Serum Adiponectin Level in Diabetic Patients with and without *Helicobacter pylori* Infection: Is There Any Difference?

**DOI:** 10.1155/2014/402685

**Published:** 2014-01-09

**Authors:** Jamshid Vafaeimanesh, Akram Heidari, Marzieh Effatpanah, Mahmoud Parham

**Affiliations:** ^1^Clinical Research Development Center, Qom University of Medical Sciences, Qom, Iran; ^2^Research Center for Medicine and Religion, Qom University of Medical Sciences, Qom, Iran; ^3^Qom University of Medical Sciences, Qom, Iran

## Abstract

*Background.* Increased insulin resistance is an extragastrointestinal manifestation of *Helicobacter pylori* (HP) infection. HP changes the level of inflammatory markers and cytokines and changes the adipocyte function by altering the adiponectin level. Given the high prevalence of HP and diabetes in our society, we evaluated the association between HP and serum adiponectin level. In this cross-sectional study, 211 diabetic patients under treatment other than insulin were studied. These patients were divided into two groups of HP+ and HP− based on their HP IgG antibody serology and their blood adiponectin levels were measured. Data was analyzed using independent *t*-test, Chi-square test, and Fisher's exact test. *Results.* Seventy-two patients with an average age of 51.56 ± 8.34 years were HP− and 139 patients with an average age of 50.35 ± 9.01 years were HP+. The mean serum adiponectin level in HP− and HP+ groups was 4.54 ± 5.43 and 5.64 ± 3.88 ng/mL, respectively. Insulin resistance degree was significantly higher in HP+ group (HP− = 3.160 ± 3.327 versus HP+ = 4.484 ± 3.781, *P* = 0.013) but no significant difference was found between the mean serum adiponectin level in HP− and HP+ groups (*P* = 0.140). *Conclusions.* Although the insulin resistance degree was significantly higher in HP+ diabetic patients, no significant relationship was found between HP infection and serum levels of adiponectin.

## 1. Introduction

Insulin resistance (IR) can be considered as a clinical state of low insulin sensitivity at one or more of the sites involving glucose metabolism in which a normal or elevated insulin level will cause an attenuated biologic response like insulin-stimulated glucose disposal [1, 2]. It is considered a common feature of type 2 diabetes mellitus and is regarded as an important mechanism in the pathogenesis of diabetes [3]. The glucose-lowering action of insulin (insulin sensitivity) shows an inverse relationship to the fasting plasma insulin. In insulin resistance state, plasma insulin levels increase to maintain a constant plasma glucose level [1]. Cardiovascular risk factors including hyperglycemia, dyslipoproteinemia, hypertension, obesity, thrombosis, and smoking are associated with increased IR risk [3]. Systemic inflammation and inflammatory cytokines may have a role in insulin resistance [4].

*Helicobacter pylori* (HP) is a human pathogen that infects the gastric mucosa and causes inflammatory process that increases in chronic gastritis, peptic ulcer, gastric lymphoma of mucosa-associated lymphoid tissue, and adenocarcinoma [5]. It is an S-shaped gram-negative microaerophilic bacillus with approximate dimensions of 0.5 × 3 micron containing multiple sheathed flagella [6]. The HP genome has 1.65 million base pairs and codes for about 1500 proteins. Of these proteins, we can refer to factors like outer membrane protein (Hop protein), urease, and vacuolating cytotoxin (Vac A) that have an essential role in virulence and colonization of HP. Moreover, most of the HP strains contain genomic fragments that encode the Cag pathogenicity island (PAI). The Cag-PAI includes a gene called Cag A that encodes parts of the Cag pathogenicity island which transfers CagA into the host cellules. Then, CagA activates a series of cellular events that are important in cell proliferation and cytokine production [7].

Based on some researches, the prevalence of HP infection in diabetics is higher than nondiabetics and its prevalence is associated with duration of diabetes [8, 9]. Also, in Agrawal et al.'s study in 2010 the prevalence of HP in diabetics was higher [10]. Although a recent cohort did not show any association between diabetes status and seropositivity to a few infective agents (including HP), HP infection increases inflammatory factors like IL-8 and TNF-*α* in diabetic patients [6].

These inflammatory factors may cause metabolic changes in these patients which may lead to insulin resistance. Different studies evaluated the association of HP with insulin resistance some of which found a positive association [11–14] while some others found no association [15–19].

On the other hand, adiponectin is a protein hormone released from adipose tissue and adipocytes. Low adiponectin level is associated with complications such as increased insulin resistance, diabetes, and metabolic syndrome [20].

Based on studies, one major cause of adiponectin level change is adipocyte changes due to inflammatory cytokines. Among them, interleukin-1 and CRP have a special place. HP causes change in these markers and cytokines due to cagA gene and, theoretically, it can change the adiponectin level by changing the adipocyte function and adiponectin level change will cause insulin resistance and eventually diabetes [21].

Given the high prevalence of HP infection and its role in insulin resistance, the aim of this study was to evaluate the association between serum adiponectin level and HP infection.

## 2. Materials and Methods

### 2.1. Patients

This cross-sectional study was carried out from May to December 2012 on diabetic patients referred to diabetes clinic of Shahid Beheshti Hospital of Qom. In case of receiving insulin, smoking, pregnancy, and a history of HP treatment (proton-pump inhibitor, H2 blocker, and bismuth), patients were excluded from the study. Then, patients were evaluated for the presence of HP infection and serum adiponectin level. After 8-hour fasting overnight, blood samples were taken at 4°C and, right after sampling, the serum was centrifuged at 2000 rpm for 15 minutes.

Anti-HP IgG antibody was measured by ELISA kit, made by Padtan Elm Co, Iran. In case of serum titer above 30 AU/mL, it was considered positive. Serum adiponectin was used by AviBion Human Adiponectin ELISA-FINLAND kit based on ng/mL. Serum insulin was measured using ELISA kit, DiaMetra Co, Italy. Multiplying the fasting glucose value (mg/dL) by serum insulin value in each person and then dividing it by 405, HOMA-IR was calculated in this study.

Data analysis was performed by SPSS version 16 using descriptive statistics, mean, standard deviation, percentage, and frequency. The analysis was performed by *t*-test, Chi-square test, and Fisher's exact test.

## 3. Results

From 211 patients enrolled in the study, 135 (64%) were female and 76 (36%) were male. The mean age and mean diabetes duration were 52.5 ± 8.8 and 7.4 ± 5.4 years, respectively.

In this study 139 patients were HP+ (65.87%) and the mean BMI in HP− and HP+ groups was 29.35 ± 4.9 and 28.79 ± 4.81, respectively, and it was not statistically significant (*P* = 0.427).

In HP− group, 47 (65.3%) patients were female and 25 (34.7%) were male and in HP+ group, 88 (63.3%) patients were female and 51 (36.7%) patients were male; HP and gender had no statistically significant difference (*P* = 0.778).

The mean abdominal circumference in HP− and HP+ groups was 101.46 ± 12.31 cm and 100.69 ± 10.82 cm, respectively, and it was not statistically significant (*P* = 0.642). Also, blood pressure was measured in both groups and had no statistically significant difference (*P* = 0.825) (Table 1).

Family history of diabetes in HP− group was positive in 53 (73.6%) and in HP+ group, 103 (74.1%) had a positive familial history of diabetes (*P* = 0.939). The mean chemical biomarkers like FBS in HP− and HP+ groups were 173.43 ± 61.31 mg/dL and 180.11 ±  64.28 mg/dL, respectively. Although it was higher in HP seropositive patients, it was not statistically significant (*P* = 0.468). Also, the mean HbA1c in HP− and HP+ groups was 8.08 ± 1.32 and 8.11 ± 1.67 mg/dL, respectively (*P* = 0.895).

The mean adiponectin level in HP− group was 4.54 ± 5.43, in HP+ group it was 5.64 ± 3.88 ng/mL, and it was not statistically different in both groups (*P* = 0.140).

Serum insulin in HP− and HP+ groups was 6.97 ± 5.64 versus 10.12 ± 7.72 (*P* = 0.002) and insulin resistance degree was significantly higher in HP+ group (HP− = 3.160 ± 3.327 versus HP+ = 4.484 ± 3.781, *P* = 0.013). The mean AST was, respectively, 20.7 ± 10.07 and 22.9 ± 13.8 mg/dL in HP− and HP+ groups (*P* = 0.244). Also the mean ALT in HP− and HP+ groups was 21.8 ± 7.7 versus 23.8 ± 14.5 (*P* = 0.274). Creatinine in HP− group was 0.91 ± 0.19 and in HP+ group it was 0.95 ± 0.29 mg/dL (*P* = 0.589).

## 4. Discussion

*Helicobacter pylori* is a human pathogen infecting the gastric mucosa which causes inflammatory process that increases in chronic gastritis, peptic ulcer, gastric lymphoma of mucosa-associated lymphoid tissue, and adenocarcinoma [5]. Aydemir et al. found an association between HP infection and metabolic syndrome. However, the mechanism of this relationship is still not well understood and the trend of studies is to determine the relationship [7].

Adiponectin is a hormone protein secreted by adipose tissue and its functions in the body include regulation of blood glucose through gluconeogenesis reduction and increased glucose uptake by cells, catabolism of fatty acids, lipid catabolism, protecting vascular endothelial dysfunction, increased insulin sensitivity, weight loss, and control of energy metabolism [22].

In most of the studies like Lihn et al.'s study, the low level of adiponectin is considered an important factor in development of metabolic syndrome and diabetes. They reported that the serum adiponectin level of patients with metabolic syndrome was significantly lower than patients without metabolic syndrome [23]. Also, Ostrowska and colleagues evaluated the serum adiponectin concentrations in patients with type 2 diabetes in 2013. They reported that adiponectin level in diabetic patients is significantly higher than that in nondiabetic ones [24].

Also, Weyer et al. reported that hypoadiponectinemia has a close association with insulin resistance and hyperinsulinemia and inflammatory factors are involved in the regulation of adiponectin [25].

Hung et al.'s study in 2008 evaluated the association of adiponectin levels with inflammatory markers, insulin resistance, and metabolic syndrome in 1094 patients 27–77 years old. They found that plasma adiponectin was a significant negative correlate of the levels of CRP and IL-6. Plasma adiponectin was also an inverse associate of HOMA-IR [26].

In our study, we found that, although HP infection increases insulin resistance in diabetic patients, this organism does not make a significant change in adiponectin level and it may aggravate insulin resistance in diabetic patients from other mechanisms. Few studies have been conducted to assess HP infection and serum adiponectin. In Ando et al.'s study in 2012, the effect of HP eradication on serum adiponectin level was evaluated in 456 patients under endoscopy for HP diagnosis. Of these patients, 338 ones had HP and underwent treatment. The serum level of adiponectin had no statistically significant difference in HP positive and negative patients but HP eradication increased the adiponectin level [27].

In another study by Hainer et al., low adiponectin conferred independent risk for impaired insulin sensitivity along with elevated WCC and HP titer [28].

## 5. Conclusion

HP seropositive diabetic patients have higher insulin resistance degrees but the adiponectin serum level in these patients has no statistically significant difference with seronegative patients and it seems that increased insulin resistance has another reason in these patients.

## Figures and Tables

**Table 1:** Characteristics and biochemical markers of the patients.

Variable	HP−	HP+	*P* value
Age (year)	51.56 ± 8.34	50.35 ± 9.01	0.120
Diabetes duration (year)	7.39 ± 5.40	7.36 ± 5.48	0.971
BMI (kg/m^2^)	29.35 ± 4.9	28.79 ± 4.81	0.427
Abdominal circumference (cm)	101.46 ± 12.31	100.69 ± 10.82	0.642
Male gender	25 (34.75%)	51 (36.7%)	0.778
Family history of diabetes	53 (73.6%)	103 (74.1%)	0.939
FBS (mg/dL)	173.43 ± 61.31	180.11 ± 64.28	0.468
HgA_1_C (mg/dL)	8.08 ± 1.32	8.11 ± 1.67	0.895
Adiponectin (ng/mL)	4.54 ± 5.43	5.64 ± 3.88	0.140
AST (mg/dL)	20.7 ± 10.07	22.9 ± 13.8	0.244
ALT (mg/dL)	21.8 ± 7.7	23.8 ± 14.5	0.274
Creatinine (mg/dL)	0.91 ± 0.19	0.95 ± 0.29	0.589
Fasting insulin level (mg/dL)	6.97 ± 5.64	10.12 ± 7.72	0.002
Insulin resistance	3.160 ± 3.327	4.484 ± 3.781	0.013
